# Supplementary material for: Association of insularity and body condition to cloacal bacteria prevalence in a small shorebird
Source: PLoS One. 2020 Aug 17;15(8):e0237369. doi: 10.1371/journal.pone.0237369 (PMC7430747; doi:10.1371/journal.pone.0237369)
Supplement: Attachment — Submitted filename: Response to reviewers.docx [file pone.0237369.s001.docx]

**Rebuttal letter**

Many thanks for your time on commenting our work. You have provided insightful comments that have made our manuscript stronger than before. This letter contains (copied and pasted) comments and suggestions regarding our manuscript and our corresponding response. The line numbers cited refer to the ‘Revised Manuscript with Track Changes’ file (as in-line changes, or as shown in the built PDF submission). In that file you will find the manuscript with all changes made, in addition to corrections of a few typos that we spotted.

The comments/suggestions are highlighted on grey colour and our response is immediately below.

**Editor comments**

Title: there is a part of the manuscript related to body condition apart from bacteria (e.g. L320-339). Therefore, I suggest to include this in the title, for example: “…prevalence and to body condition in a…”

You have raised a good point. We agree with this change. Now the title reads:

‘Association of insularity and body condition to cloacal bacteria prevalence in a small shorebird’

L99: The first prediction has been properly explained before. However, the other three predictions are not previously introduced. For example, we do not know why we could expect differences between sexes (prediction ii), and although it is intuitive, there are no arguments in the Introduction to expect the negative relationship between bacteria and body condition (prediction iii). Finally, concerning to prediction iv, the relationship between the immunity and habitat (island vs. mainland) has been explained, but this is not the case with the body condition. Although the immunity is part of the general condition, here you refer specifically to the scale mass index, which could be positively or negatively related with the immune response.

Therefore, before list the predictions, all of them should be properly introduced.

After your comment we realised the gap in the information we had provided. We have now added a couple of lines to provide this information. You can find this throughout the aims section, **in lines: 91-108.**

L189 and Fig. 3: In the text, authors say that they used as body condition the scale mass index. However, in the Fig. 3, it seems like if you used the body mass, and actually, it is expressed in grams (g). Please, clarify this point.

We appreciate this observation, however, after a detailed revision, we believe we indeed used the scaled mass index correctly.

As cited in the text, we used the equation proposed by Peig & green (2009):

Scaled mass index:

$$\hat{M}_{i}=M_{i}\left[ \frac{L_{0}}{L_{i}} \right]^{b_{\mathrm{SMA}}}$$

Where $\hat{M}_{i}$ is the allometrically corrected body mass for individual $i$ when the body measure is standardized $L_{0}$, to an arbitrary value of $L$. $M_{i}$ and $L_{i}$ are the body mass and the body (in grams) linear measurement (here, wing length) of individual $i$, respectively; $b_{\mathrm{SMA}}$ is the scaling exponent estimated.

When examining the equation, it is possible to know that the resulting adjusted mass of each individual will maintain its unit (g). In fact, usually the difference between and individual’s body mass and the scaled mass index is minimal, just a few grams.

We modified **line 256** in order to make the figure 3 more clear and more consistent with the description of the scaled mass index in the methods section.

Also, we expand a bit further on this topic in the very last answer in this document.

Peig J, Green AJ. New perspectives for estimating body condition from mass/length data: the scaled mass index as an alternative method. Oikos. 2009; 118:1883–1891. doi: 10.1111/j.1600-0706.2009.17643.x

L136: Change sampled to sampling.

L251: Change to “reason”

L351: change to “bacterial infection”

All these minor changes have been adopted. Many thanks for spotting them.

**Additional requirements**

2. In your Methods section, please provide additional location information of the study sites, including geographic coordinates for the data set if available.

Coordinates of geographical locations added in methods section.

3. We note that you have stated that you will provide repository information for your data at acceptance. Should your manuscript be accepted for publication, we will hold it until you provide the relevant accession numbers or DOIs necessary to access your data. If you wish to make changes to your Data Availability statement, please describe these changes in your cover letter and we will update your Data Availability statement to reflect the information you provide.

In accordance to the previously stated, we have submitted to a public repository the full dataset and R code used to conduct this study. Link: <https://doi.org/10.5061/dryad.9ghx3fffg>

4. We note that Figure 1 in your submission contain map images which may be copyrighted. All PLOS content is published under the Creative Commons Attribution License (CC BY 4.0), which means that the manuscript, images, and Supporting Information files will be freely available online, and any third party is permitted to access, download, copy, distribute, and use these materials in any way, even commercially, with proper attribution. For these reasons, we cannot publish previously copyrighted maps or satellite images created using proprietary data, such as Google software (Google Maps, Street View, and Earth). For more information, see our copyright guidelines: http://journals.plos.org/plosone/s/licenses-and-copyright.

This will not be an issue because the map in Figure 1 was done from scratch.

**Reviewers comments**

My main question while reading this paper regards as to why the authors choose throughout the paper present these bacterial genera as established pathogens? The authors seem to imply it is presumed these organism cause harm and disease to the host. However, it is well known that these bacterial genera may often present no adverse host effects in birds. This has been extensively studied in chickens where they are often found as commensals. The authors seem aware of this fact, since they write in line 38: “Campylobacter and Salmonella are gram-negative bacteria from the Enterobacteriaceae family and often found as commensal microbiota in avian hosts [36, 37].” Seeing how the authors may pick up different species from the studied genera, how are they able to tell that the obtained organisms are in fact pathogenic in these birds?

This is indeed and interesting point. While in poultry these bacteria are often commensals, in wild birds their status is rather variable and experiments are necessary to evaluate pathogenicity. Additionally, in many cases the impact of bacteria on their hosts is condition dependent, and well feed individuals do not experience any negative effect while other individuals may suffer fitness reduction.

A number of infection experiments show that wild bird do present a negative response to for example, *Camplylobacter jejuni*, suffering weight loss and mounting an immune response (Waldeström et al. 2010; Atterby et al. 2018). While *Salmonella enterica* has been a well-known cause of mortality in wild birds (Lawson et al. 2014).

To date, it is unknown whether Kentish plover has species-specific *Campylobacter* or *Salmonella* strains and to what degree these strains are commensals or can harm host health.

Based on this evidence, we believe our manuscript has been well focussed. We nevertheless have added a couple of sentences to make this clearer throughout the text. **Lines 93-95, 157-161, 346-348**.

Many thanks for your comment.

Waldenström J, et al. Campylobacter jejuni colonization in wild birds: results from an infection experiment. PLoS One. 2010; 5:e9082. doi: 10.1371/journal.pone.0009082

Atterby C, et al. The potential of isolation source to predict colonization in avian hosts: A case study in Campylobacter jejuni strains from three bird species. Front Microbiol. 2018; 9:591. doi: 10.3389/fmicb.2018.00591

Lawson B, et al. Epidemiological evidence that garden birds are a source of human Salmonellosis in England and Wales. PLoS One. 2014; 9(2):e88968. doi:10.1371/journal.pone.0088968

Seeing how these bacteria are often acquired through sexual transmission (line 144), is it not of importance to account for host age? One would assume younger individuals have experienced fewer sexual encounters and less possibilities for transmission. I understand it may be difficult to assess host age in these birds, but even so it might deserve a mention somewhere.

Unfortunately, we don’t have detailed information of individual’s age other than they are more than one year old. It is true that we can expect prevalence to increase with age, but this prediction is true for sexually transmitted parasites but also for vector borne parasites or directly transmitted parasites.

It is worth noting that the age/infection association of sexually transmitted infections is not as simple as it seems. Several studies in humans (where it has been more studied) show that the highest prevalences of infection often occur in young adults (20-30 y), not in older people (e.g. LaMontagne et al. 2003; Turner et al. 2011). To our knowledge, there is not previous work investigating the effect of age on sexually transmitted diseases in birds. A notable previous study on lizards showed that multiple mates was positively correlated with cloacal diversity, but interestingly, diversity decreased with age (White et al. 2011).

Given that we did not include this variable in our study, we believe that is not convenient to expand on this topic that seems to be controversial and that also has to be put in the context of island vs mainland.

LaMontagne DS, Fine DN, Marrazzo JM. Chlamydia trachomatis infection in asymptomatic men. Am J Prev Med. 2003;24(1):36-42. doi:10.1016/s0749-3797(02)00573-1

Turner AN, et al. Prevalence of gonococcal and chlamydial infection in 2009 in 2 populations in a midwestern city. J Natl Med Assoc. 2011;103(4):313-321. doi:10.1016/s0027-9684(15)30312-6

White J, Richard M, Massot M, Meylan S (2011) Cloacal Bacterial Diversity Increases with Multiple Mates: Evidence of Sexual Transmission in Female Common Lizards. PLoS ONE 6(7): e22339. doi:10.1371/journal.pone.0022339

L147 brings up Chlamydia psittaci, but there’s no evidence in the current study that this is the bacterial species that is being sampled.

Thank you for noticing this. We mentioned *Chlamydia psittaci* in that specific part just as an example of a well-known pathogenic species of *Chlamydia* in birds. In fact, earlier in that section (**lines 159, 162**), we mention other examples for the other bacteria genera that we studied: ‘Pathogenic strains like *Campylobacter lari* or *Salmonella typhimurium* have been associated with gastrointestinal disease in poultry and in wild birds’.

Furthermore, elsewhere in the manuscript, particularly in the Discussion section (**lines 307-310**) we emphasise our comments only on *Chlamydia* as a genus.

I’m missing some details in the Methods how the three bacterial genera were amplified. I’m aware the authors refer to other studies, but it would be good if they can add some further details so a reader does not have to dig up and read through an additional three papers just to figure out what the authors sequenced.

We have added this missing information. **Lines 177-182**.

Many thanks for the suggestion.

• L258: “salinity is a well-known inhibitor of bacteria growth” Yes, but what is the effect of salinity on these three specific genera that is sampled? That’s the important point to discuss.

In fact the papers cited were specific on the effect of salinity on the bacteria genera studied. We have modified this section slightly to emphasise on this. **Line 284.**

Question: Is higher body mass always regarded as having better body condition?

Good question. The answer is that is not always the case.

For example, in a bird population with a mean body mass of 41.41 g and mean wing length of 110.1 mm, if we have two individuals A and B:

| Bird | Body mass (g) | Wing length (mm) | Body condition index (g) |
| --- | --- | --- | --- |
| A | 43.06 | 114 | 41.87 |
| B | 43.30 | 116.5 | 41.38 |

From looking just at the body masses of bird A and B we may say that bird A is in worse condition than Bird B. But, after correcting for size, we know that bird A is actually in better body condition than bird B. This is because bird B is apparently a larger bird than bird A based on wing length.
